# Closing Address of Dr. Geo. B. Wood, to the Class of the Medical Department of the University of Penn., Feb. 29th, 1860

**Published:** 1860-05

**Authors:** 


*Closing Address of Dr. Geo. B. Wood, to the Class of the Medical Department of the University of Penn., Feb. 29th,* 1860.

[The following is the substance of Dr. Wood's closing remarks to the medical class of the University of Pennsylvania, when retiring from his professorship in that institution.—For the report we are indebted to Dr. J. Solis Cohen, of this city.—EDS.]

You have now heard, probably, the last lecture of the last course of lectures I shall ever deliver upon medical subjects. You are aware that I propose resigning my position in this school. I announced my intention to do so, at a somewhat early period, in order that the authorities might have time to satisfy themselves in the choice of my successor.

Perhaps you may think it due from me to give some reasons for this course. In the first place, I will mention that I have now been lecturing for at least forty years, without a single intermission during that time, and frequently, for many years, both in the winter and the summer seasons.

Beginning as a private lecturer, I afterwards entered as a Professor, the College of Pharmacy, where I continued for nearly fifteen years, and the remaining twenty-five years have occupied a position in this school.

This length of service, perhaps, entitles me to rest. But there are other considerations. I am advancing in age.—Though I have not yet reached the period of life at which mental imbecility and infancy occurs, yet the time will, before long come. It is true, there are some favored individuals, who go on to extreme old age and maintain their faculties to the last, as is fully exemplified in the present Prime Minister of England. But this is not the general rule. Much more commonly, the faculties begin to fail anterior to the 70th year, and I have no right to suppose that I shall be an exception to that rule. You know very well that when the mind begins to fail, the individual is scarcely conscious of it himself. He does not appreciate the full deficiency of his own powers, and there is danger that he may be overtaken by it while in the discharge of his public duties, without being himself aware of his insufficiency. There is thus danger that he may become a burthen on the institution to which he is attached. I do not indeed know that I have not myself begun to enter upon this period. I cannot be, however, far advanced in it, and I wish to secure myself against the chance of getting into this false position. Besides, I desire to enjoy a period of leisure before the time comes when I shall cease to have pleasure in life, while I can still appreciate and enjoy the results of observations in foreign countries. In the course of a few years, I feel that it would be no longer possible for me to have this sort of enjoyment. It is, therefore, I think, advisable for me to withdraw from my present duties, a little while before I might feel myself absolutely bound to do so, from consideration for the interests of the institution. But I do not wish to be considered as intending to abandon the profession of medicine. When abroad, it is my intention to pay special attention to medical subjects; and on my return, I hope to be able to occupy myself with the general interests of medicine, and as far as capacity may remain to supply it, for the good of a profession to which I have been so long attached.

Having said so much about myself, I will for a few minutes ask attention to the class.

I have said on several former occasions, when addressing the pupils of this school, that I have noticed a gradual improvement in the character of the several classes which have come under my notice. This may be considered as a natural result of the position of our school. I think we may claim that our classes have been somewhat select; and that they have a tendency to become more and more so with the extension of medical education in various parts of the country. I assure you, gentlemen, I do not think the present class forms an exception to the general rule of progress. I have had the opportunity of examining a large number of you every week, and, certainly, I have never before been so well satisfied with the answers that have been made me. Your deportment generally, through the winter, has been all that we could wish. In my own relations with you, there has been nothing upon which I can look back with an unpleasant reflection; not a shadow rests on my remembrance of our intercourse. Your respectful attention and personal courtesy have been very grateful to me, I assure you; and I thank you most heartily for all your kindness.

May I, as a man old enough certainly to be the father of any of you, give you some little advice as to your future course of life? Do not suppose that your education is completed. You have laid only the foundation, and erected the skeleton and frame-work which you are to fill up. Go home, therefore, with the intention of prosecuting your studies vigorously, and do not give out even when business may hereafter press upon you. Do not get into the habit of acting solely and not learning; for we are receiving constant accessions to our medical information and knowledge; and it requires the constant attention of the practitioner to keep himself up with the level of the times. It is not only in reference to your medical knowledge I wish to impress upon you the value of certain courses of action. I wish you always to entertain a due opinion and feeling as to the dignity and importance of your profession. Consider that the reputation of the profession is more or less involved with your own; that you may, by your own conduct, increase or diminish the estimation in which it will be held; and let this be a strong inducement to regulate your whole course in accordance with the rules of honor and morality. Cultivate carefully all the exteriors which characterize the gentleman; but especially cultivate your moral sense, looking not only to future prosperity in this world, but also to your state in that which is to come. I am sure there is no one of you who, at his last hour, will regret that he has paid some attention to the advice which I now give you. Long experience in life authorizes me, perhaps, to offer some lessons for your use, guidance and assistance in the future. This consideration has been one of my inducements for publishing in a single volume the introductory lectures I have at various times delivered, and for placing a copy of the book in possession of each member of the class. I do not wish you to read these lectures regularly through. Put the book on your shelves. Once in a while, when you feel occasion for counsel, take it down; you will find lessons applicable to most of the circumstances in which you may be placed, and when you consult its pages, may I not ask of you to give one thought to your old preceptor?

I have little more to say. I shall probably have the opportunity, before we part finally, to take each one of you by the hand, perhaps more than once, but as the class of the session now ending, I must bid you farewell. May heaven, gentlemen, shower its choicest blessings upon you.—*Medical Reporter.*

